# Supplementary material for: Perceived neighborhood social cohesion and functional disability among older adults: The moderating roles of sex, physical activity, and multi-morbidity
Source: PLoS One. 2024 Jan 31;19(1):e0293016. doi: 10.1371/journal.pone.0293016 (PMC10830004; doi:10.1371/journal.pone.0293016)
Supplement: S1 Table — (DOCX) [file pone.0293016.s002.docx]

S1 Table. **Univariate and bivariate analysis of independent variables and functional disability**

| Independent variables | Overall | Functional disability | | p-value |
| --- | --- | --- | --- | --- |
|  | N (%) | No disability, N (%) | With disability, N (%) |  |
| **Age** (Mean, SD) | 57.6±16.7 | 55.0±16.0 | 74.1±12.2 | <0.001 |
| **Gender** |  |  |  | <0.01 |
| Male | 1,826 (41.1) | 1,658 (41.7) | 168 (35.5) |  |
| Female | 2,620 (58.9) | 2,315 (58.3) | 305 (64.5) |  |
| **Marital status** |  |  |  | <0.001 |
| Never married | 416 (9.36) | 409 (10.3) | 7 (1.48) |  |
| Married/cohabiting | 2,555 (57.5) | 2,366 (59.6) | 189 (40.0) |  |
| Separated/divorce | 499 (11.2) | 442 (11.1) | 57 (12.1) |  |
| Widowed | 976 (21.9) | 756 (19.0) | 220 (46.5) |  |
| **Location of residence** |  |  |  | 0.331 |
| Rural | 2,624 (59.0) | 2,335 (58.8) | 289 (61.1) |  |
| Urban | 1822 (41.0) | 1,638 (41.2) | 184 (38.9) |  |
| **Education** |  |  |  | 0.056 |
| Less than primary school | 610 (23.6) | 559 (23.0) | 51 (32.5) |  |
| Primary education completed | 664 (25.7) | 629 (25.9) | 35 (22.3) |  |
| Senior high completed | 1,168 (45.2) | 1,106 (45.6) | 62 (39.5) |  |
| University degree/post | 142 (5.50) | 133 (5.48) | 9 (5.73) |  |
| **Health status** |  |  |  | <0.001 |
| Good | 627 (18.4) | 625 (19.8) | 2 (0.76) |  |
| Moderate | 2,448 (71.7) | 2,366 (75.1) | 82 (31.1) |  |
| Bad | 341 (9.98) | 161 (5.11) | 180 (68.2) |  |
| **Multimorbidity** |  |  |  | <0.001 |
| No morbidity | 2,390(53.8) | 2,217 (55.8) | 173 (36.6) |  |
| Only one morbidity | 517 (11.6) | 430 (10.8) | 87 (18.4) |  |
| 2 or more morbidities | 1,539 (34.6) | 1,326 (33.4) | 213 (45.0) |  |
| **Physical activity (PA)** |  |  |  |  |
| **Vigorous-intensity activity** |  |  |  | <0.001 |
| Yes | 1,346 (30.5) | 1,324 (33.6) | 22 (4.69) |  |
| No | 3,069 (69.5) | 2,622 (66.5) | 447 (95.3) |  |
| **Moderate-intensity activity** |  |  |  | <0.001 |
| Yes | 2,576 (58.4) | 2,489 (63.1) | 87 (18.6) |  |
| No | 1,839 (41.7) | 1,457 (36.9) | 382 (81.5) |  |
| **Walk** |  |  |  | <0.001 |
| Yes | 2,878 (65.2) | 2,684 (68.0) | 194 (41.4) |  |
| No | 1,537 (34.8) | 1,262 (32.0) | 275 (58.6) |  |
| **PA (Overall)** |  |  |  | <0.001 |
| Yes | 3,392 (76.3) | 3,182 (80.1) | 210 (44.4) |  |
| No | 1,054 (23.7) | 791 (19.9) | 263 (55.6) |  |
| **Perceived Neighbourhood social cohesion** |  |  |  |  |
| *Perceived Community level participation (Mean, SD)* | 24.1±7.79 | 24.8±7.71 | 18.3±5.69 | <0.001 |
| *Perceived Trust (Mean SD)* | 10.1±3.16 | 10.2±3.16 | 9.86±3.06 | <0.05 |
| *Perceived safety (Mean, SD)* | 8.25±1.56 | 8.25±1.55 | 8.26±1.61 | 0.99 |
| Perceived Neighbourhood social cohesion (Overall) | 42.5±9.31 | 43.3±9.30 | 36.7±6.85 | <0.001 |
| **Functional disability** |  |  |  |  |
| No | 3,973 (89.4) | - | - | - |
| Yes | 473 (10.6) | - | - | - |
