# Supplementary material for: Perceived neighborhood social cohesion and functional disability among older adults: The moderating roles of sex, physical activity, and multi-morbidity
Source: PLoS One. 2024 Jan 31;19(1):e0293016. doi: 10.1371/journal.pone.0293016 (PMC10830004; doi:10.1371/journal.pone.0293016)
Supplement: S2 Table — (DOCX) [file pone.0293016.s003.docx]

**S2 Table.** **Relationship between Perceived neighbourhood social cohesion (overall) and functional disability adjusted for confounders (N=4,446)**

|  | [1] | [2] | [3] | [4] | [5] | [6] | [7] |
| --- | --- | --- | --- | --- | --- | --- | --- |
| **Neighbourhood social cohesion (NSC)** | 0.94 (0.93, 0.94)*** | 0.94 (0.93, 0.95)*** | 0.94 (0.93, 0.95)*** | 0.94 (0.93, 0.95)*** | 0.94 (0.93, 0.95)*** | 0.95 (0.94, 0.96)*** | 0.96 (0.0.95, 0.97)*** |
| **Age** |  | 1.10 (1.09, 1.11)*** |  |  |  |  | 1.09 (1.08, 1.10)*** |
| **Gender** |  |  |  |  |  |  |  |
| Male |  |  | 1 |  |  |  | 1 |
| Female |  |  | 1.13 (0.92, 1.40) |  |  |  | 1.37 (1.02, 1.83)* |
| **Marital status** |  |  |  |  |  |  |  |
| Never married |  |  |  | 0.17 (0.07, 0.39)*** |  |  | 0.41 (0.17, 1.00) |
| Married/cohabiting |  |  |  | 1 |  |  | 1 |
| Separated/divorce |  |  |  | 1.52 (1.09, 2.11)** |  |  | 1.41 (0.96, 2.06) |
| Widowed |  |  |  | 3.3.10 (2.48,3.88)*** |  |  | 1.18 (0.87, 1.60) |
| **Multimorbidity** |  |  |  |  |  |  |  |
| No morbidity |  |  |  |  | 1 |  | 1 |
| Any one morbidity |  |  |  |  | 2.29 (1.71, 3.08)*** |  | 0.94 (0.66, 1.32) |
| 2 or more morbidities |  |  |  |  | 1.93 (1.54, 2.41)*** |  | 1.27 (0.98, 1.65) |
| **Physical activity** |  |  |  |  |  | 0.26 (0.21, 0.32)*** | 0.45 (0.23, 0.86)** |

***Notes: Health status was removed leaving multi-morbidity because the final model was better without it. .Model 1- NSC and functional disability; Model 2 – NSC, functional disability and age; Model 3- NSC, functional disability and gender; Model 4 - NSC, functional disability and marital status; Model 5 - NSC, functional disability and multimorbidity; Model 6- NSC, functional disability and physical activity; model 7 - NSC, functional disability, age, gender, marital status, multi-morbidity and physical activity. ***, **, * denote significant levels at 1%, 5% and 10%, respectively.***
