# Supplementary material for: Perceived neighborhood social cohesion and functional disability among older adults: The moderating roles of sex, physical activity, and multi-morbidity
Source: PLoS One. 2024 Jan 31;19(1):e0293016. doi: 10.1371/journal.pone.0293016 (PMC10830004; doi:10.1371/journal.pone.0293016)
Supplement: S3 Table — (DOCX) [file pone.0293016.s004.docx]

**S3 Table.** **Effects of confounding on the relationship between measures of perceived neighbourhood social cohesion and functional disability**

|  | [1] | [2] | [3] | [4] | [5] | [6] | [7] | [8] | [9] | [10] | [11] | [12] | [13] | [14] | [15] | [16] | [17] | [18] | [19] | [20] | [21] |
| --- | --- | --- | --- | --- | --- | --- | --- | --- | --- | --- | --- | --- | --- | --- | --- | --- | --- | --- | --- | --- | --- |
| Perceived Community Participation | 0.94 (0.94, 0.95)*** | 0.95 (0.94, 0.96)*** | 0.94 (0.94, 0.95)*** | 0.94 (0.94, 0.95)*** | 0.94 (0.94, 0.95)*** | 0.96 (0.95, 0.96)*** | 0.96 (0.95, 0.97)*** |  |  |  |  |  |  |  |  |  |  |  |  |  |  |
| Perceived Trust |  |  |  |  |  |  |  | 1.00 (0.99, 1.00)* | 0.99 (0.99, 1.00)** | 1.00 (0.99, 1.00)* | 1.00 (0.99, 1.00) | 1.00 (0.99, 1.00) | 0.99 (0.99, 1.00)** | 0.99 (0.99, 1.00)** |  |  |  |  |  |  |  |
| Perceived safety |  |  |  |  |  |  |  |  |  |  |  |  |  |  | 1.00 (0.99, 1.01) | 1.00 (0.99, 1.01) | 1.00 (0.99, 101) | 1.00 (1.00, 1.01) | 1.00 (0.99, 1.01) | 1.00 (1.00, 1.01) | 1.00 (1.00, 1.01) |

*Note: Model 1- Community participation and functional disability; Model 2 – Community participation, functional disability and age; Model 3- Community participation, functional disability and sex; Model 4 - Community participation, functional disability and marital status; model 5 - Community participation, functional disability and multimorbidity; model 6 - Community participation, functional disability and physical activity; Model 7 - Community participation, functional disability, age, sex, marital status, multi-morbidity, and physical activity; Model 8- Trust and functional disability; Model 9 – Trust, functional disability and age; Model 10- Trust, functional disability and sex; Model 11 - Trust functional disability and marital status; model 12 - Trust functional disability and multimorbidity; Model 13 - Trust, functional disability and physical activity; Model 14 - Trust, functional disability, age, sex, marital status, multi-morbidity, and physical activity; Model 15- Safety and functional disability; Model 16 – Safety, functional disability and age; Model 17- Safety, functional disability and sex; Model 18 - Safety, functional disability and marital status; Model 19 - Safety, functional disability and multimorbidity; Model 20 - Safety functional disability and physical activity; Model 21 - Safety, functional disability, age, sex, marital status, multi-morbidity, and physical activity. ***, **, * denote significant levels at 1%, 5% and 10%.*
