# Supplementary material for: Perceived neighborhood social cohesion and functional disability among older adults: The moderating roles of sex, physical activity, and multi-morbidity
Source: PLoS One. 2024 Jan 31;19(1):e0293016. doi: 10.1371/journal.pone.0293016 (PMC10830004; doi:10.1371/journal.pone.0293016)
Supplement: S4 Table — (DOCX) [file pone.0293016.s005.docx]

**S4 Table. Sex, multimorbidity and physical activity moderation on perceived neighbourhood social cohesion association with functional disability**

|  | [1] | [2] | [3] | [4] | [5] | [6] | [7] |
| --- | --- | --- | --- | --- | --- | --- | --- |
| Perceived neighbourhood social cohesion (Overall) | 0.94 (0.93, 0.94)*** |  |  |  |  |  |  |
| **Sex** |  |  |  |  |  |  |  |
| Male |  | 1 |  |  |  |  |  |
| Female |  | 1.30 (1.07, 1.59)* |  |  |  |  |  |
| **Multimorbidity** |  |  |  |  |  |  |  |
| No morbidity |  |  | 1 |  |  |  |  |
| Any one morbidity |  |  | 2.59 (0.96, 3.42)*** |  |  |  |  |
| 2 or more morbidities |  |  | 2.06 (1.67, 2.54)*** |  |  |  |  |
| **Physical activity** |  |  |  |  |  |  |  |
| Yes |  |  |  | 0.20 (0.16, 0.24)*** |  |  |  |
| No |  |  |  | 1 |  |  |  |
| **NSC*Sex** |  |  |  |  |  |  |  |
| Male |  |  |  |  | 1 |  |  |
| NSC*female |  |  |  |  | 0.99 (0.97, 1.01) |  |  |
| **NSC*Multi-morbidity** |  |  |  |  |  |  |  |
| No morbidity |  |  |  |  |  | 1 |  |
| NSC*Any one morbidity |  |  |  |  |  | 0.99 (0.97, 1.02) |  |
| NSC*2 or more morbidities |  |  |  |  |  | 0.99 (0.97, 1.01) |  |
| **NSC*physical activity** |  |  |  |  |  |  |  |
| NSC*yes |  |  |  |  |  |  | 0.99 (0.97, 1.00) |
| No |  |  |  |  |  |  | 1 |

***Model 1 – Model 4 – Odds ratio between each variable and functional disability; Model 5 – Sex interaction on association between perceived neighbourhood social cohesion and functional disability; Model 6 – Multimorbidity interaction on the association between perceived neighbourhood social cohesion and functional disability; Model 7 – Physical activity interaction on the association between perceived neighbourhood social cohesion and functional disability.***
