# Supplementary material for: Perceived neighborhood social cohesion and functional disability among older adults: The moderating roles of sex, physical activity, and multi-morbidity
Source: PLoS One. 2024 Jan 31;19(1):e0293016. doi: 10.1371/journal.pone.0293016 (PMC10830004; doi:10.1371/journal.pone.0293016)
Supplement: S5 Table — (DOCX) [file pone.0293016.s006.docx]

**S5 Table. Sex, multimorbidity and physical activity moderation on perceived community-level participation association with functional disability**

|  | [1] | [2] | [3] | [4] | [5] | [6] | [7] |
| --- | --- | --- | --- | --- | --- | --- | --- |
| Community level participation (CLP) | 0.94 (0.94, 0.95)*** |  |  |  |  |  |  |
| **Gender** |  |  |  |  |  |  |  |
| Male |  | 1 |  |  |  |  |  |
| Female |  | 1.30 (1.07, 1.59)* |  |  |  |  |  |
| **Multimorbidity** |  |  |  | , |  |  |  |
| No morbidity |  |  | 1 |  |  |  |  |
| Any one morbidity |  |  | 2.59 (0.96, 3.42)*** |  |  |  |  |
| 2 or more morbidities |  |  | 2.06 (1.67, 2.54)*** |  |  |  |  |
| **Physical activity** |  |  |  |  |  |  |  |
| Yes |  |  |  | 0.20 (0.16, 0.24)*** |  |  |  |
| No |  |  |  | 1 |  |  |  |
| **CLP*Sex** |  |  |  |  |  |  |  |
| Male |  |  |  |  | 1 |  |  |
| CLP*female |  |  |  |  | 0.99 (0.98, 1.01) |  |  |
| **CLP*Multi-morbidity** |  |  |  |  |  |  |  |
| No morbidity |  |  |  |  |  | 1 |  |
| CLP*Any one morbidity |  |  |  |  |  | 0.99 (0.97, 1.01) |  |
| CLP *2 or more morbidities |  |  |  |  |  | 1.00 (0.98, 1.01) |  |
| CLP ***physical activity** |  |  |  |  |  |  |  |
| CLP *yes |  |  |  |  |  |  | 0.98 (0.96, 0.99)** |
| No |  |  |  |  |  |  |  |

***Model 1 – Model 4 – Odds ratio between each variable and functional disability; Model 5 – gender interaction on association between perceived community-level participation and functional disability; Model 6 – Multimorbidity interaction on the association between perceived community-level participation and functional disability; Model 7 – Physical activity interaction on the association between perceived community-level participation and functional disability.***
