# Supplementary material for: Perceived neighborhood social cohesion and functional disability among older adults: The moderating roles of sex, physical activity, and multi-morbidity
Source: PLoS One. 2024 Jan 31;19(1):e0293016. doi: 10.1371/journal.pone.0293016 (PMC10830004; doi:10.1371/journal.pone.0293016)
Supplement: S1 Appendix — (DOCX) [file pone.0293016.s007.docx]

**S1 Appendix. List of the 12 variables included in the WHODAS score and cut points**

| In the last 30 days how much difficulty do you have in: | |
| --- | --- |
| …learning a new task, for example, learning how to get to a new place? |  |
| …making new friendships or maintaining current friendships? |  |
| …dealing with strangers? |  |
| …standing for long periods, such as 30 min? |  |
| …taking care of your household responsibilities? |  |
| …joining community activities (for example, festivities, religious or other activities) in the same way as anyone else can? |  |
| …concentrating on doing something for 10 min? |  |
| …walking long distance, such as one kilometer? |  |
| …bathing/washing your whole body? |  |
| …getting dressed? |  |
| …performing your day to day work? |  |
| In the last 30 days, how much have you been emotionally affected by your health condition(s)? |  |

None = 0; Mild = 1; Moderate = 2; Severe = 3; Extreme/cannot = 4
