# Supplementary material for: Perceived neighborhood social cohesion and functional disability among older adults: The moderating roles of sex, physical activity, and multi-morbidity
Source: PLoS One. 2024 Jan 31;19(1):e0293016. doi: 10.1371/journal.pone.0293016 (PMC10830004; doi:10.1371/journal.pone.0293016)
Supplement: S2 Appendix — (DOCX) [file pone.0293016.s008.docx]

**S2 Appendix. Perceived Neighborhood social cohesion measure**

|  |  |  |  |  |  |
| --- | --- | --- | --- | --- | --- |
| 1. **Community involvement (9 questions)** | Never | Once or twice per year | Once or twice per month | Once or twice per week | Daily |
| How often in the last 12 months have you ………… |  |  |  |  |  |
| … attended any public meetings in which there was discussion of local or school affairs? | 1 | 2 | 3 | 4 | 5 |
| … met personally with someone you consider to be a community leader? | 1 | 2 | 3 | 4 | 5 |
| …attended any group, club, society, unio or organizational meeting? | 1 | 2 | 3 | 4 | 5 |
| … worked with other people in your neighborhood to fix or improve something? | 1 | 2 | 3 | 4 | 5 |
| …. had friends over to your home? | 1 | 2 | 3 | 4 | 5 |
| … being in the home of someone who lives in a different neighborhood than you do or had them in your home? | 1 | 2 | 3 | 4 | 5 |
| … socialized with coworkers outside of work? | 1 | 2 | 3 | 4 | 5 |
| …. attended religious services (not including weddings and funerals)? | 1 | 2 | 3 | 4 | 5 |
| …. gotten out of the house your dwelling to attend social meetings, activities, programs or events or to visit friends or relatives. | 1 | 2 | 3 | 4 | 5 |
| 1. **Perceived Trust** |  |  |  |  |  |
| Next, we did like to know how much you trust different groups of people: | To a very great extent | To a great extent | Neither great nor small extent | To a small extent | To a very small extent |
| First, think about people in your neighborhood. Would you say that you can trust them……? | 1 | 2 | 3 | 4 | 5 |
| Now, think about people whom you work with. Generally speaking, would you say that you can trust them….? | 1 | 2 | 3 | 4 | 5 |
| And how about strangers? Generally speaking, would you say that you can trust them ….? | 1 | 2 | 3 | 4 | 5 |
| 1. **Perceived safety** (1-completely safe, very safe, moderately safe, slightly safe, not safe)- |  |  |  |  |  |
| Now we have a few questions about safety in the area where you live | Completely safe | Very safe | Moderately safe | Slightly safe | Not safe at all |
| In general, how safe you feel when walking down your street alone after dark? | 1 | 2 | 3 | 4 | 5 |
| How safe do you feel when walking down your street alone after dark? | 1 | 2 | 3 | 4 | 5 |
