# Supplementary material for: Perceived neighborhood social cohesion and functional disability among older adults: The moderating roles of sex, physical activity, and multi-morbidity
Source: PLoS One. 2024 Jan 31;19(1):e0293016. doi: 10.1371/journal.pone.0293016 (PMC10830004; doi:10.1371/journal.pone.0293016)
Supplement: Attachment — Submitted filename: 2023-05-24-1940.pdf [file pone.0293016.s010.pdf]

Manuscript_similarity.docx
May 24, 2023
5080 words / 29746 characters

Manuscript_similarity.docx

Sources Overview

34%
OVERALL SIMILARITY

#	Source	Type	Similarity
1	www.ncbi.nlm.nih.gov	INTERNET	11%
2	journals.plos.org	INTERNET	5%
3	eprints.soton.ac.uk	INTERNET	2%
4	repository.essex.ac.uk	INTERNET	2%
5	jscholarship.library.jhu.edu	INTERNET	2%
6	Kofi Awuviry-Newton, Dinah Amoah, Meredith Tavener, Adjeiwa Akosua Afram, Pablo Villalobos Dintrans, Julie Byles, Paul Kowal. "Food Insecurity and Functional Disability among Older Adults in Ghan...	CROSSREF	1%
7	www.researchgate.net	INTERNET	1%
8	doczz.net	INTERNET	<1%
9	www.who.int	INTERNET	<1%
10	ugspace.ug.edu.gh	INTERNET	<1%
11	Eric S Kim, Armani M Hawes, Jacqui Smith. "Perceived neighbourhood social cohesion and myocardial infarction", Journal of Epidemiology and Community Health, 2014	CROSSREF	<1%
12	apps.who.int	INTERNET	<1%
13	nigerianstat.gov.ng	INTERNET	<1%
14	Cassandra L. Hua, J. Scott Brown. "Childhood Socioeconomic Status and Physical Activity in Later Life: The Role of Perceived Neighborhood Cohesion and Wealth in Adulthood", Journal of Applied Ge...	CROSSREF	<1%
15	Eric S. Kim, Ying Chen, Ichiro Kawachi, Tyler J. VanderWeele. "Perceived neighborhood social cohesion and subsequent health and well-being in older adults: An outcome-wide longitudinal approach", ...	CROSSREF	<1%
16	link.springer.com	INTERNET	<1%
17	Eric S. Kim, Nansook Park, Christopher Peterson. "Perceived neighborhood social cohesion and stroke", Social Science & Medicine, 2013	CROSSREF	<1%
18	www.duo.uio.no	INTERNET	<1%
19	Kaston D. Anderson-Carpenter, Hannah M. Sauter, José G. Luiggi-Hernández, Paige E. Haight. "Associations between Perceived Homophobia, Community Connectedness, and Having a Primary Care ...	CROSSREF	<1%
20	www.science.gov	INTERNET	<1%
21	Milagros Ruiz, Sofia Malyutina, Andrzej Pajak, Magdalena Kozela, Ruzena Kubinova, Martin Bobak. "Congruent relations between perceived neighbourhood social cohesion and depressive symptoms ...	CROSSREF	<1%
22	www.hec.unil.ch	INTERNET	<1%
23	"Handbook of Health and Well-Being", Springer Science and Business Media LLC, 2022	CROSSREF	<1%
24	Terrence D. Hill, Ha Ngoc Trinh, Ming Wen, Lauren Hale. "Perceived neighborhood safety and sleep quality: a global analysis of six countries", Sleep Medicine, 2016	CROSSREF	<1%
25	E. Greenglass, L. Fiksenbaum, J. Eaton. "The relationship between coping, social support, functional disability and depression in the elderly", Anxiety, Stress & Coping, 2006	CROSSREF	<1%
26	Erasmus University of Rotterdam on 2021-08-13	SUBMITTED WORKS	<1%

27 Fei Wang, Weidi Qin, Jiao Yu. "Neighborhood Social Cohesion and Mobility Limitations Among Community-dwelling Older Americans: The Mediating Roles of Depressive Symptoms and Mastery", The I...
CROSSREF
<1%

28 Hill, Terrence D., Ha Ngoc Trinh, Ming Wen, and Lauren Hale. "Perceived neighborhood safety and sleep quality: a global analysis of six countries", Sleep Medicine, 2016.
CROSSREF
<1%

29 Xiangli Chen, Veronica Muller, Beatrice Lee, Jia-Rung Wu, Kanako Iwanaga, Deborah Lee. "Community Participation as Both a Mediator and Moderator for the Relationship Between Functional Disabili...
CROSSREF
<1%

30 "SCIENTIFIC ABSTRACTS", Journal of General Internal Medicine, 4/2006
CROSSREF
<1%

31 Gloria Chepngeno-Langat, Jane C. Falkingham, Nyovani J. Madise, Maria Evandrou. "Concern About HIV and AIDS Among Older People in the Slums of Nairobi, Kenya", Risk Analysis, 2012
CROSSREF
<1%

32 London School of Hygiene and Tropical Medicine on 2018-09-04
SUBMITTED WORKS
<1%

33 Yeonwoo Kim, Ahyoung Lee, Catherine Cubbin. "Effect of Social Environments on Cardiovascular Disease in the United States", Journal of the American Heart Association, 2022
CROSSREF
<1%

34 f1000research.com
INTERNET
<1%

35 research.bangor.ac.uk
INTERNET
<1%

36 research.library.mun.ca
INTERNET
<1%

37 Boqin Xie, Chenjuan Ma, Junqiao Wang. "Independent and Combined Relationships of Perceived Neighborhood Social Cohesion and Physical Frailty on Functional Disability in Community-Dwelling Ol...
CROSSREF
<1%

38 Kim Nichols Dauner, Neil A. Wilmot, Jennifer F. Schultz. "Investigating the temporal relationship between individual-level social capital and health in fragile families", BMC Public Health, 2015
CROSSREF
<1%

39 R.B. Biritwum, N. Minicuci, A.E. Yawson, O. Theou et al. "Prevalence of and factors associated with frailty and disability in older adults from China, Ghana, India, Mexico, Russia and South Africa", Mat...
CROSSREF
<1%

40 University of Johannsburg on 2013-09-19
SUBMITTED WORKS
<1%

41 University of Leeds on 2014-09-10
SUBMITTED WORKS
<1%

42 Duke Appiah, Sujata Luitel, Nayeli Fuentes, Chike C. Nwabuo. "Perceived neighborhood social cohesion and the 10-year risk of cardiovascular disease in low- and middle-income countries: The World H...
CROSSREF
<1%

43 International Perspectives on Aging, 2015.
CROSSREF
<1%

44 Kwasi Adjepong Darkwah, Samuel Iddi, Justice Nonvignon, Moses Aikins. "Characterization of functional disability among older adults in Ghana: A multi-level analysis of the study on global ageing an...
CROSSREF
<1%

45 Mengyun Luo, Philayrath Phongsavan, Adrian Bauman, Joel Negin, Zhiruo Zhang, Ding Ding. "Correlates of Domain-Specific Physical Activity Among Older Adults in Six Low- to Middle-Income Countri...
CROSSREF
<1%

Excluded search repositories:

- None

Excluded from document:

- None

Excluded sources:

- None

Perceived neighborhood social cohesion and functional disability among older adults in Ghana: the moderating roles of sex, physical activity, and multi-morbidity

Abstract

Though the Ghanaian social structure is largely communal in several of its social life and social spaces, the extent to which cohesive neighbourhood affects functional ability of older persons and the moderating factors of the relationship, are unknown in Ghana. This study examines the moderating roles of sex, multimorbidity, and physical activity on the association between neighbourhood social cohesion and functional disability among older people in Ghana. A cross-sectional study of 4,446 people—50 years and older—from WHO's Study on global AGEing and adult health Ghana Wave 2. Functional disability —WHO Disability Assessment Schedule 2.0—and neighbourhood social cohesion measured with community-level participation, trust and safety were studied. Generalised Logistic regressions with interactional tests were used to examine the associations. A more socially cohesive neighbourhood was significantly associated with a lower functional disability among older people (OR = 0.94, 95%CI: 0.93, 0.94). A similar relationship was found for community-level participation (aOR=0.94, 95%CI: 0.94, 0.95) and perceived trust (aOR=1.00, 95%CI: 0.99, 1.00). Community-level participation is associated with a lower functional disability among older people who were physically active (aOR=0.98, 95%CI: 0.96, 0.99). Among the three individual-level measures of neighbourhood social cohesion, physical activity only moderated the association between community-level participation and functional disability. Community-level participation, along with physical activity, may be relevant in improving functional ability among older people. The results highlight the usefulness of policy to ensure a more socially cohesive neighbourhood for older people in Ghana to improve their quality of life.

Introduction

As the proportion and number of older people grow globally including Ghana [1], opportunities emerge at understanding how neighborhood social cohesion can strengthen older people's functional abilities.. The term perceived neighborhood social cohesion - defined in this study - refers to a sense of trust, safety, and participation among people who live in the same place [2-4]. Often neighborhood social cohesion is considered as an important element in public health due to its influential role on the wellbeing older people [5]. As adults age, incidence of chronic illness and difficulty in daily activities increase, older people may respond to the decline in physical health by using social resources, including connections, trust, and the social bonds [4]. When perceptions of trust, safety and participation prevail in a community where older adults are residents, they will freely involve in all activities, which will eventually strengthen their functional capacities. Owing to this reasoning and evidence, perceived neighborhood social cohesion may associate with functional disability - the difficulty individuals experience in engaging in activities of daily living such as bathing, using public transport, caring for households and toileting [6, 7] among older people in Ghana.

Although life expectancy of Ghanaian older people is increasing—52 years in 2005 vs 58 years in 2019; 2.81% rise [8]—, functional disability among older people in Ghana is noticeable. For instance, a nationally representative study comparing functional disability score among six countries (China, Mexico, Ghana, South Africa, India, Russia) that participated in the global study on AGEing and adults' health (SAGE) project reported a lowest in China, highest in India with Ghana and South Africa recording the next higher score [9]. About 90% of older people in Ghana reported a difficulty across domains functioning assessment including cognition, mobility, self-care, getting along with people, engagement in household responsibility and participation in

society [9]. A recent qualitative study exploring older people' lived experiences of their functional disability revealed that they feel anxious and restricted in being productive [10]. Given this significant functional disability prevalence among older people in Ghana, they may rely on informal social networks and other organized events to overcome daily life demands, including dealing with functional disability. In this context, cohesive neighborhoods could be an important channel to influence functional disability among older people. Though the Ghanaian social structure is largely communal in several of its social life and social spaces [11], the extent to which cohesive neighbourhood associate with functional disability of older persons and the nature of the relationship, is unexplored in Ghana.

Significant evidence on the relationship between neighbourhood social cohesion and the three measures (perceived safety, trust and participation) and functional disability exist in western countries. These studies revealed that older people who report higher levels of perceived social cohesion have a lower chance of developing stroke, and myocardial infarction [12, 13]. On the other hand, lower perceived social cohesion through living alone is associated with isolation, reduced social activities and interpersonal ties [4, 14], increased likelihood of developing functional disability [15, 16], thereby acting as protective factor against functional disability. In the study by Stephens, Allen [17], a greater accessibility of neighborhoods and higher level of trust among neighbours associate with better mental health.

In low-and middle-income countries including Ghana evidence on neighbourhood social cohesion and its measures, and functional disability among older people is scant. Rahman and Singh [18] used data from six countries of WHO SAGE project on functional disability and social cohesion and reported that functional disability is associated with lower social cohesion, with social cohesion being highest among males, rural dwellers, currently married, currently working,

better educated, and higher on the socio-economic ladder across all six countries [18]. For example, participating in social and group activities that promote health behaviors such as physical activity and healthy diet habits may delay the development of functional disability [19, 20]. In Ghana, studies on associations between functional disability and other variables such as food insecurity [21, 22], and social isolation, neighbourhood walkability and loneliness [23, 24], physical activity[25], and long-term care [26] exist with limited understanding of how perceived neighborhood social cohesion and its measures associate with functional disability.

Known factors from western literature moderating the association between perceived neighborhood social cohesion and functional disability include physical activity [19, 20, 27], chronic conditions such as stroke and heart attack [12] and, age and sex [18, 28]. In Ghana, very little is known about the moderating variables of the association between perceived neighborhood social cohesion and functional disability.

In this study, we examined the potential effect of perceived neighborhood social cohesion on functional disability, particularly identifying how the association between perceived community-level participation, trust, and safety within communities and functional disability are moderated by sex, multi-morbidity, and physical activity in Ghana. The findings will serve as a baseline for policymakers and researchers in Ghana, but also to extract important lessons regarding social policies for ageing populations in low- and middle-income countries.

Methods

Study sample

We used data from the Study on global AGEing and adult health (SAGE) Ghana Wave 2 conducted between 2014/2015. The actual number of participants who participated in SAGE Ghana Wave 2 was 4,704, however only 4,446 responded to questions related to the independent variable -

functional disability. SAGE is a Multi-Country (Ghana, South Africa, China, India, Mexico, and Russia) longitudinal study that employed multistage cluster sampling strategies [29]. The University of Ghana Medical School through the Department of Community Health, and in collaboration with the World Health Organization (WHO), implemented the SAGE Wave 2 in Ghana.

Variables

Functional disability

Functional disability was defined using the 12-item version of the WHO Disability Assessment Schedule (WHODAS 2.0), which classifies responses into five disability categories: none, mild, moderate, severe, and extremely severe (see Appendix A). In its full version, the WHODAS 2.0 contains 12 questions from six domains: cognition, mobility, self-care, getting along, life activities, and participation in society [30] Supplement 1 contains the questions included in the analysis. WHODAS 2.0 was scored on a scale of 0 to 100 determining the severity of the disability [9, 31, 32]. Participants scoring <90.18% were denoted as "no disability" and participants who score >=90.18% were denoted as "with a disability."

Perceived neighborhood social cohesion

Perceived neighborhood social cohesion was measured from three domains namely perceived community participation, perceived trust, and perceived safety used in WHO SAGE 2 [29]. Nine questions each with a 5-response category were used to measure older people's perception of their participation in their communities (See Appendix B for details). In the resulting scale for community-level participation, from 9 to 45, with higher values representing higher levels of community participation. The reliability coefficient was 0.87. Three questions were used to measure perceptions of feeling of trust among participants in the community. Five responses

namely "to a very great extent" 1, "to a great extent" 2, neither great nor small extent" 3, "to a small extent" 4, and "to a very small extent" 5 (see Appendix B). These responses were reversed coded so that a higher number represented higher neighbours trust level. For instance, "to a very great extent" was coded "5" whereas the "to a very small extent was coded "1". The responses were highly reliable at alpha=0.889. Perceived safety was measured with two variables with 5 response categories with 1 representing completely safe, 2 representing very safe, 3 moderately safe, 4 slightly safe and 5 representing not safe at all. These responses were reversed coded so that a higher number represented higher neighbours perceived feeling of safety. For instance, "completely safe" was coded "5" whereas the "not safe at all "1". The reliability coefficient was 0.847, with values ranging from 2 to 10.

The three individual-level variables 1) Perceived community-level participation, 2) perceived trust and 3) perceived safety were put together using *transformation scale* to measure perceived neighborhood social cohesion. The higher the score the higher levels of perceived neighborhood social cohesion. The values range from 14 to 70 with higher values indicating a more cohesive neighborhood. The Cronbach's alpha of 0.839 represented a high reliability.

Sex

The question what your sex is dichotomised as Male=1 and Female=2 was used.

Physical activity

Physical activity was measured with three separate items including vigorous activity, moderate activity, and walking. Vigorous-intensity activity was measured by the question *"Does your work involve vigorous-intensity activity that causes large increases in breathing or heart rate, [like heavy lifting, digging, or chopping wood] for at least 10 minutes continuously (Yes/No)?* The question *"Does your work involve moderate-intensity activity that causes small increases in*

breathing or heart rate [such as brisk walking, carrying light loads, cleaning, cooking, or washing clothes] for at least 10 minutes continuously?" (Yes/No) was used to measure work-related moderate-intensity activity. We used the question *"Do you walk or use a bicycle (pedal cycle) for at least 10 minutes continuously to get to and from places?" (Yes/ No)* to measure older people' engagement in walk.

Additionally, these three measures were scored and aggregated into two response categories; *yes* (engages in at least one of the three measures of physical activity) and *no* (engages in none of the three measures). The Cronbach's α of the three physical activity items combined was 61%.

Multimorbidity

The question *"Have you ever been diagnosed with/told you have ...? (Yes/No)* was used to identify the presence of each of the 10 chronic conditions including stroke, hypertension, depression, diabetes, angina, arthritis, chronic lung disease, asthma, cataract, and oral health among older people. Responses were combined and a variable capturing the presence of different conditions was generated to measure multimorbidity (1=*no condition, 2=one chronic condition and 3=at least two condition*).

Covariates

Sociodemographic and health confounding variables included in the analysis were age (continuous), marital status (1=never married, 2=married/cohabiting, 3=separated/divorced, 4=widowed), education (1=less than primary school, 2=primary education completed, 3=senior high completed, 4=university degree/post), location of residence (1=rural, 2=urban) and self-reported health status (1=good, 2=moderate, 3=bad).

Data analysis

First, descriptive statistics such as frequency, percentages, and means and standard deviations were used to describe the variables in the study. Second, bivariate analyses were performed through chi-square, Fisher's test, and t-test to establish relationships between functional disability and neighbourhoods' social cohesion and its domains. Finally, and multivariate logistic regression were performed to estimate the odds ratios (Crudes and adjusted) and 95% confidence intervals (CI) for the associations between perceived neighbourhood social cohesion and functional disability. A moderation analysis (multiplicative terms) of sex, multimorbidity and physical activity in the relationship were estimated at 0.05.

Results

Characteristics of Study Participants

The characteristics of study participants are described in Table 1. The mean age of participants with functional disability was approximately 74 years, with higher proportion of females reporting higher functional disability compared to the proportion of males (64.5% vs 35.5%). A high prevalence of functional disability was found among widowed (46.5%), rural dwellers (61.1%), senior high school leavers (39.5%), those who reported health as bad (68.2%) and lived with at least two chronic conditions (45.0%). A high prevalence of older people who reported absence physical activity engagement of any kind suffered functional disability (55.6%) compared to those who are physically active (55.6% vs 44.4%). Older people with no functional disability had a higher mean perceived community level participation score compared with older people with functional disability (24.8 vs. 18.3, P<0.001).

[Insert Table 1 here]

Neighbourhood Social Cohesion and Functional Disability

In the unadjusted model, overall perceived neighbourhood social cohesion was statistically associated with functional disability (OR=0.94, 95%CI: 0.93, 0.94). When adjusted for potential confounders including age, gender and marital status, the strength of association existing between overall perceived neighbourhood social cohesion and functional disability was still significant and of similar magnitude (OR=0.94, 95%CI: 0.93, 0.95) (Table 2).

[Insert Table 2 here]

In Table 3, the associations between perceived community-level participation level and perceived trust with functional disability were significant after adjusting for potential confounders. However, the adjusted association between perceived safety and functional disability (after controlling for potential confounders) shows to be statistically insignificant.

[Insert Table 3 here]

Table 4 shows the interactional effect of sex, multimorbidity and physical activity on the neighborhood association with functional disability. None of the interactional variables studied moderated the association between overall neighborhood social cohesion and functional disability.

[Insert Table 4 here]

Among the three measures of perceived neighborhood social cohesion studied, the association between perceived community-level participation and functional disability was significant with the moderators (OR=0.94, 95%CI: 0.94, 0.95). Even among the three moderators, only physical activity moderated the association between perceived community-level participation and functional disability. That is, older people who were involved in community activities and report engaging in physical activity were 2% less likely to experience functional disability (OR, 0.98, CI: 0.98, 0.99) (see Table 5).

[Insert Table 5 here]

Discussion

Evidence on the effect of sex, physical activity, and multi-mobility on the association between perceived social cohesion and functional disability is least established in the gerontological literature from low- and middle- income countries. To contribute to addressing this knowledge gap, the aim of this study was to determine the moderating role of sex, physical activity, and multi-morbidity on the association between perceived neighbourhood social cohesion and functional disability among older people in Ghana. The important findings for policy and practice implications are discussed.

The current study finding that a more perceived socially cohesive neighbourhood was associated with a lower functional disability among older people confirms available studies in other low- and middle-income countries [33-35]. Adding to this evidence, in Japan, previous gerontological studies have reported that social cohesive neighbourhoods reduce functional disability [35]. In a related study, Aida, Kondo [34] reported that higher incidence of functional disability is linked to lower community social capital among women in Japan. Three important reasons may explain the relationship between social cohesion and functional disability among older people in the literature. Firstly, with high socially cohesive neighborhoods, older people may have higher odds of getting access to social support when they have health problems [36]; with access to social support, they can seek early health treatment(s) to prevent the onset of functional disability [16]. Secondly, social cohesion reduces the development of functional disability through social networking and group activities which result in positive health behaviour such as physical activity and healthy diets [37, 38]. Lastly, increased social cohesion is linked to improved mental and physical wellbeing which lessen the functional disability [16, 39]. Our results thus suggest that older people with higher socially cohesive neighbourhoods tend to demonstrate better functional and psychological health. Given the significant effect of social cohesion on functional disability, ensuring socially cohesive neighborhood is likely to improve functional status of older people and also reduce the risk of functional disability

associated with ageing [16]. This implies that to ensure improved health of older people, social cohesion such as social connections and trust should be considered as a health priority [35]. Our results further imply that to lessen functional disability, community-based measures which foster social capital may be important [34].

It is important to highlight that perceived trust and perceived community-level participation, which are part of the framework for measuring socially cohesive neighborhoods were both associated with functional disability. Interestingly, the study revealed that increased in social trust and community-level participation reduces functional disability. This finding underscores the need to promote social trust and community participation in old age to reduce the risks of development of functional disability at the community levels. Clear evidenced based policy initiatives are required to be implemented to foster social trust building at the community level.

The findings from this study are consistent with the observations made by previous gerontological studies conducted elsewhere [40, 41]. Corroborating the present findings, a study conducted in China reiterates how increase in social participation in old age predicts lower risk of the developing functional disability [42]. This finding further affirms Fujihara, Miyaguni [41] assertion older people with increased level of community participation (such as sports) are less likely to report better functional health. This result further reinforces Chen, Min [40] finding that community participation moderates the relationship between functional satisfaction among life satisfaction.

Our finding that increased level of community-level participation lowers the risk of functional disability in old age may be attributed to three possible reasons. In the first place, older people who participate actively and more in community events such as communal labour and other outdoor activities are less likely to be sedentary and as a result have better functional health. Secondly, older people with increased community-level participation have lower odds of being homebound compared with those with low level of community participation thereby reducing

their financial disability [41]. Thirdly, social participation enhances access to health-relevant information in old age, which is important to promoting functional health. Lastly, social participation enables older people to stay active (such as dressing each day to leave home) and these daily functions help to improve their functional health [43].

The above reasons are grounded based our finding that physical activity moderated the association between community-level participation and functional disability. These findings highlight several important policy implications. First, preventive programme and/or policy to improve functional ability among older people should encourage older people to participate frequently and more actively in social events at the community levels. Second, healthcare providers rendering care to older people need to gain a better understanding of the relevance of socially cohesive neighborhoods in improving functional ability in old age. Third, to improve functional ability of older people, diverse indicators for measuring socially cohesive neighborhood (such as social capital) should be considered [39]. This is because several factors such as trust and community-level participation as a dimension of socially cohesive neighbourhoods have proven to reduce the risk of functional disability in old age. Lastly, there is the need to promote physical activity in old age for a desired functional health and quality of life.

The study has some limitations that need to be considered. The extent of the analysis is limited by the availability of data. Issues regarding internal validity—e.g., due to other variables that could be mediating the relationship between social cohesion and functional disability—and external validity that need to be considered. However, results are in line with other studies showing a promising research and policy area that has not been extensively explored, especially in Ghana and other low- and middle-income contexts. Data from the SAGE study Wave 2 collected during 2014/2015 could be dated; however, given the current trends in population ageing and functional disability, it is expected that results have even more relevance today.

Our findings arise as important for policymakers, since they highlight the relevance of social policies that help building social cohesion not just as important per se and to improve community wellbeing but also as a strategy to address the expected increase in long-term care needs coming from population ageing and the rise in the prevalence of functional disability. The results are important for Ghana and other low- and middle-income countries since can be seen as an efficient policy—freeing two birds with one key—for addressing the pressing social security demands in these countries.

Conclusion

Findings from this nationally representative study demonstrated the importance of a socially cohesive neighbourhood in reducing the risk of functional disability among older people, through physical activity in their long-term care. The findings have implications for policy makers to ensure social cohesiveness is improved by fostering the establishment of social support groups and local community network groups in any health and social care systems that seek to address an aspect of the long-term care needs of older people. Setting up community centres where older adults could meet their contemporaries and engage in life-enhancing activities, such as, exercises will improve their physical health. Such facility will offer older adults the opportunity for social interaction and reduce or eliminate the loneliness and its associated functional disability experienced by older people. Healthcare providers should also emphasise on the need for older people to be physically active while family friendly relationships should be strengthened. A holistic approach is needed to ensure a socially cohesiveness community rather than a single entity. Further study is warranted to establish the nature and trajectory of community-level participation that help reduce functional disability.

Table 1. **Univariate and bivariate analysis of independent variables and functional disability**

Independent variables	Overall N (%)	Functional disability		p-value
		No disability, N (%)	With disability, N (%)	
**Age** (Mean, SD)	57.6±16.7	55.0±16.0	74.1±12.2	<0.001

**Gender**				<0.01
Male	1,826 (41.1)	1,658 (41.7)	168 (35.5)	
Female	2,620 (58.9)	2,315 (58.3)	305 (64.5)	
**Marital status**				<0.001
Never married	416 (9.36)	409 (10.3)	7 (1.48)	
Married/cohabiting	2,555 (57.5)	2,366 (59.6)	189 (40.0)	
Separated/divorce	499 (11.2)	442 (11.1)	57 (12.1)	
Widowed	976 (21.9)	756 (19.0)	220 (46.5)	
**Location of residence**				0.331
Rural	2,624 (59.0)	2,335 (58.8)	289 (61.1)	
Urban	1822 (41.0)	1,638 (41.2)	184 (38.9)	
**Education**				0.056
Less than primary school	610 (23.6)	559 (23.0)	51 (32.5)	
Primary education completed	664 (25.7)	629 (25.9)	35 (22.3)	
Senior high completed	1,168 (45.2)	1,106 (45.6)	62 (39.5)	
University degree/post	142 (5.50)	133 (5.48)	9 (5.73)	
**Health status**				<0.001
Good	627 (18.4)	625 (19.8)	2 (0.76)	
Moderate	2,448 (71.7)	2,366 (75.1)	82 (31.1)	
Bad	341 (9.98)	161 (5.11)	180 (68.2)	
**Multimorbidity**				<0.001
No morbidity	2,390(53.8)	2,217 (55.8)	173 (36.6)	
Only one morbidity	517 (11.6)	430 (10.8)	87 (18.4)	
2 or more morbidities	1,539 (34.6)	1,326 (33.4)	213 (45.0)	
**Physical activity (PA)**				
**Vigorous-intensity activity**				<0.001
Yes	1,346 (30.5)	1,324 (33.6)	22 (4.69)	
No	3,069 (69.5)	2,622 (66.5)	447 (95.3)	
**Moderate-intensity activity**				<0.001
Yes	2,576 (58.4)	2,489 (63.1)	87 (18.6)	
No	1,839 (41.7)	1,457 (36.9)	382 (81.5)	
**Walk**				<0.001
Yes	2,878 (65.2)	2,684 (68.0)	194 (41.4)	
No	1,537 (34.8)	1,262 (32.0)	275 (58.6)	
**PA (Overall)**				<0.001
Yes	3,392 (76.3)	3,182 (80.1)	210 (44.4)	
No	1,054 (23.7)	791 (19.9)	263 (55.6)	
**Perceived Neighbourhood social cohesion**				
Perceived Community level participation (Mean, SD)	24.1±7.79	24.8±7.71	18.3±5.69	<0.001
Perceived Trust (Mean SD)	10.1±3.16	10.2±3.16	9.86±3.06	<0.05

Perceived safety (Mean, SD)	8.25±1.56	8.25±1.55	8.26±1.61	0.99
Perceived Neighbourhood social cohesion (Overall)	42.5±9.31	43.3±9.30	36.7±6.85	<0.001
**Functional disability**				
No	3,973 (89.4)	-	-	-
Yes	473 (10.6)	-	-	-

Table 2. **Relationship between Perceived neighbourhood social cohesion (overall) and functional disability adjusted for confound[illegible]**

(N=4,446)

	[1]	[2]	[3]	[4]	[5]	[6]	[7[illegible]
**Neighbourhood social cohesion (NSC)**	0.94 (0.93, 0.94)***	0.94 (0.93, 0.95)***	0.94 (0.93, 0.95)***	0.94 (0.93, 0.95)***	0.94 (0.93, 0.95)***	0.95 (0.94, 0.96)***	0.96 (0.0[illegible] 0.97)***
**Age**		1.10 (1.09, 1.11)***					1.09 (1.0[illegible] 1.10)***
**Gender**							
Male			1				1
Female			1.13 (0.92, 1.40)				1.37 (1.0[illegible] 1.83)*
**Marital status**							
Never married				0.17 (0.07, 0.39)***			0.41 (0.1[illegible]
Married/cohabiting				1			1
Separated/divorce				1.52 (1.09, 2.11)**			1.41 (0.9[illegible]
Widowed				3.3.10 (2.48,3.88)***			1.18 (0.8[illegible]
**Multimorbidity**							
No morbidity					1		1
Any one morbidity					2.29 (1.71, 3.08)***		0.94 (0.6[illegible]
2 or more morbidities					1.93 (1.54, 2.41)***		1.27 (0.9[illegible]
**Physical activity**						0.26 (0.21, 0.32)***	0.45 (0.2[illegible] 0.86)**

Notes: Health status was removed leaving multi-morbidity because the final model was better without it. .Model 1- NSC and functional disability; Model 2 – NSC, functional disability and age; Model [illegible] functional disability and gender; Model 4 - NSC, functional disability and marital status; Model 5 - NSC, functional disability and multimorbidity; Model 6- NSC, functional disability and physical ac[illegible] 7 - NSC, functional disability, age, gender, marital status, multi-morbidity and physical activity. ***, **, * denote significant levels at 1%, 5% and 10%, respectively.

Table 3. **Effects of confounding on the relationship between measures of perceived neighbourhood social cohesion and functional disability**

	[1]	[2]	[3]	[4]	[5]	[6]	[7]	[8]	[9]	[10]	[11]	[12]	[13]	[14]	[15]	[16]	[17]	[18]
Perceived Community Participation	0.94 (0.94, 0.95)***	0.95 (0.94, 0.96)***	0.94 (0.94, 0.95)***	0.94 (0.94, 0.95)***	0.94 (0.94, 0.95)***	0.96 (0.95, 0.96)***	0.96 (0.95, 0.97)***											
Perceived Trust								1.00 (0.99, 1.00)*	0.99 (0.99, 1.00)**	1.00 (0.99, 1.00)*	1.00 (0.99, 1.00)	1.00 (0.99, 1.00)	0.99 (0.99, 1.00)**	0.99 (0.99, 1.00)**				
Perceived safety															1.00 (0.99, 1.01)	1.00 (0.99, 1.01)	1.00 (0.99, 101)	1.00 (1.00, 1.01)

Note: Model 1- Community participation and functional disability; Model 2 – Community participation, functional disability and age; Model 3- Community participation, functional dis[illegible] *sex; Model 4 - Community participation, functional disability and marital status; model 5 - Community participation, functional disability and multimorbidity; model 6 - Community pa*[illegible] *functional disability and physical activity; Model 7 - Community participation, functional disability, age, sex, marital status, multi-morbidity, and physical activity; Model 8- Trust and* [illegible] *disability; Model 9 – Trust, functional disability and age; Model 10- Trust, functional disability and sex; Model 11 - Trust functional disability and marital status; model 12 - Trust func*[illegible] *disability and multimorbidity; Model 13 - Trust, functional disability and physical activity; Model 14 - Trust, functional disability, age, sex, marital status, multi-morbidity, and physica*[illegible] *Model 15- Safety and functional disability; Model 16 – Safety, functional disability and age; Model 17- Safety, functional disability and sex; Model 18 - Safety, functional disability and* [illegible] *status; Model 19 - Safety, functional disability and multimorbidity; Model 20 - Safety functional disability and physical activity; Model 21 - Safety, functional disability, age, sex, marit*[illegible] *multi-morbidity, and physical activity. ***, **, * denote significant levels at 1%, 5% and 10%.*

Table 4. Sex, multimorbidity and physical activity moderation on perceived neighbourhood social cohesion association with functional disab

	[1]	[2]	[3]	[4]	[5]	[6]	[7]
Perceived neighbourhood social cohesion (Overall)	0.94 (0.93, 0.94)***						
**Sex**							
Male		1					
Female		1.30 (1.07, 1.59)*					
**Multimorbidity**							
No morbidity			1				
Any one morbidity			2.59 (0.96, 3.42)***				
2 or more morbidities			2.06 (1.67, 2.54)***				
**Physical activity**							
Yes				0.20 (0.16, 0.24)***			
No				1			
**NSC*Sex**							
Male					1		
NSC*female					0.99 (0.97, 1.01)		
**NSC*Multi-morbidity**							
No morbidity						1	
NSC*Any one morbidity						0.99 (0.97, 1.02)	
NSC*2 or more morbidities						0.99 (0.97, 1.01)	
**NSC*physical activity**							
NSC*yes							0.9[illegible] 1.0[illegible]
No							1

Model 1 – Model 4 – Odds ratio between each variable and functional disability; Model 5 – Sex interaction on association between perceived neighbourhood social cohesion and disability; Model 6 – Multimorbidity interaction on the association between perceived neighbourhood social cohesion and functional disability; Model 7 – Physical activity intera the association between perceived neighbourhood social cohesion and functional disability.

Table 5. Sex, multimorbidity and physical activity moderation on perceived community-level participation association with functional disab

	[1]	[2]	[3]	[4]	[5]	[6]	[7]
Community level participation (CLP)	0.94 (0.94, 0.95)***						
**Gender**							
Male		1					
Female		1.30 (1.07, 1.59)*					
**Multimorbidity**				,			
No morbidity			1				
Any one morbidity			2.59 (0.96, 3.42)***				
2 or more morbidities			2.06 (1.67, 2.54)***				
**Physical activity**							
Yes				0.20 (0.16, 0.24)***			
No				1			
**CLP*Sex**							
Male					1		
CLP*female					0.99 (0.98, 1.01)		
**CLP*Multi-morbidity**							
No morbidity						1	
CLP*Any one morbidity						0.99 (0.97, 1.01)	
CLP *2 or more morbidities						1.00 (0.98, 1.01)	
CLP ***physical activity**							
CLP *yes							0.98 (0.96, 0.99)**

No	

Model 1 – Model 4 – Odds ratio between each variable and functional disability; Model 5 – gender interaction on association between perceived community-level participation a[illegible] functional disability; Model 6 – Multimorbidity interaction on the association between perceived community-level participation and functional disability; Model 7 – Physical acti[illegible] interaction on the association between perceived community-level participation and functional disability.

Appendix

Appendix A

Supplement 1. List of the 12 variables included in the WHODAS score and cut points

In the last 30 days how much difficulty do you have in:	
...learning a new task, for example, learning how to get to a new place?	
...making new friendships or maintaining current friendships?	
...dealing with strangers?	
...standing for long periods, such as 30 min?	
...taking care of your household responsibilities?	
...joining community activities (for example, festivities, religious or other activities) in the same way as anyone else can?	
...concentrating on doing something for 10 min?	
...walking long distance, such as one kilometer?	
...bathing/washing your whole body?	
...getting dressed?	
...performing your day to day work?	
In the last 30 days, how much have you been emotionally affected by your health condition(s)?	

None = 0; Mild = 1; Moderate = 2; Severe = 3; Extreme/cannot = 4

Appendix B

Perceived Neighborhood social cohesion					
a. Community involvement (9 questions)	Never	Once or twice per year	Once or twice per month	Once or twice per week	Daily
How often in the last 12 months have you ………					
… attended any public meetings in which there was discussion of local or school affairs?	1	2	3	4	5
… met personally with someone you consider to be a community leader?	1	2	3	4	5
…attended any group, club, society, unio or organizational meeting?	1	2	3	4	5
… worked with other people in your neighborhood to fix or improve something?	1	2	3	4	5
…. had friends over to your home?	1	2	3	4	5
… being in the home of someone who lives in a different neighborhood than you do or had them in your home?	1	2	3	4	5
… socialized with coworkers outside of work?	1	2	3	4	5
…attended religious services (not including weddings and funerals)?	1	2	3	4	5
…. gotten out of the house your dwelling to attend social meetings, activities, programs or events or to visit friends or relatives.	1	2	3	4	5
b. Perceived Trust					
Next, we did like to know how much you trust different groups of people:	To a very great extent	To a great extent	Neither great nor small extent	To a small extent	To a very small extent
First, think about people in your neighborhood. Would you say that you can trust them……?	1	2	3	4	5
Now, think about people whom you work with. Generally speaking, would you say that you can trust them….?	1	2	3	4	5

And how about strangers? Generally speaking, would you say that you can trust them ....?	1	2	3	4	5
c. ***Perceived safety*** *(1-completely safe, very safe, moderately safe, slightly safe, not safe)-*					
Now we have a few questions about safety in the area where you live	Completely safe	Very safe	Moderately safe	Slightly safe	Not safe at all
In general, how safe you feel when walking down your street alone after dark?	1	2	3	4	5
How safe do you feel when walking down your street alone after dark?	1	2	3	4	5
