# Supplementary material for: Perceived neighborhood social cohesion and functional disability among older adults: The moderating roles of sex, physical activity, and multi-morbidity
Source: PLoS One. 2024 Jan 31;19(1):e0293016. doi: 10.1371/journal.pone.0293016 (PMC10830004; doi:10.1371/journal.pone.0293016)
Supplement: Attachment — Submitted filename: Response to reviewers.docx [file pone.0293016.s011.docx]

**Response to reviewers**

Dear editor and reviewers,

We appreciate the privilege extended to us to revise the manuscript. We acknowledge that the comments raised are relevant and they have substantially improved the manuscript.

1. **Please ensure that your manuscript meets PLOS ONE's style requirements, including those for file naming.**

**Response**

We have formatted the manuscript to conform to the Plos One’s style requirements.

2. **Thank you for stating the following financial disclosure:
      "The funders had no role in study design, data collection and analysis, decision to publish, or preparation of the manuscript."**

**Response**

This was an error and so we have rectified this error. The funding declaration now reads as “The authors received no specific funding for this work.”

3. **In your Data Availability statement, you have not specified where the minimal data set underlying the results described in your manuscript can be found. PLOS defines a study's minimal data set as the underlying data used to reach the conclusions drawn in the manuscript and any additional data required to replicate the reported study findings in their entirety. All PLOS journals require that the minimal data set be made fully available. For more information about our data policy, please see** [**http://journals.plos.org/plosone/s/data-availability**](http://journals.plos.org/plosone/s/data-availability)**.**

**Response**

Authors have provided the minimal dataset underlying this manuscript. We have uploaded this as a supplementary document.

4. **Please include your full ethics statement in the ‘Methods’ section of your manuscript file. In your statement, please include the full name of the IRB or ethics committee who approved or waived your study, as well as whether you obtained informed written or verbal consent. If consent was waived for your study, please include this information in your statement as well.**

Response

We have provided the ethics statement in the “Methods” section, with a mention of the ethics committee and reflection on participants consent. It now states as “The World Health Organisation Ethical Research Committee provided ethical approval for this study (#ID3925). Participants provided a written and/or verbal consent for the study” under “ethical consideration”.

5. **Please review your reference list to ensure that it is complete and correct. If you have cited papers that have been retracted, please include the rationale for doing so in the manuscript text or remove these references and replace them with relevant current references. Any changes to the reference list should be mentioned in the rebuttal letter that accompanies your revised manuscript. If you need to cite a retracted article, indicate the article’s retracted status in the References list and also include a citation and full reference for the retraction notice.**

**Response**

All references have been cross-checked. All citations have their corresponding list of references towards the end of the manuscript.

6. Please include captions for your Supporting Information files at the end of your manuscript, and update any in-text citations to match accordingly. Please see our Supporting Information guidelines for more information: <http://journals.plos.org/plosone/s/supporting-information>.

**Response**

Captions for all supporting documents have been included at the end of the manuscript.

7. Thanks for submitting your manuscript to PLOS ONE. Your manuscript's similarity is currently at 34% (without the reference list and author information), which is above the minimum value allowed by PLOS ONE. Ideally, your manuscript should be 10% or less. Can I ask you to edit your work carefully to bring its similarity to the acceptable level? The file showing your similarity index is attached. Please note that your manuscript has not been peer-reviewed, so this decision is only formative and applies to the manuscript's originality.

**Response**

I have paraphrased all areas. This are evident on the manuscript.
