# Supplementary material for: Perceived neighborhood social cohesion and functional disability among older adults: The moderating roles of sex, physical activity, and multi-morbidity
Source: PLoS One. 2024 Jan 31;19(1):e0293016. doi: 10.1371/journal.pone.0293016 (PMC10830004; doi:10.1371/journal.pone.0293016)
Supplement: Attachment — Submitted filename: Review comments.docx [file pone.0293016.s012.docx]

**General comments**

There were no line numbers in the document. Thus, it made it quite difficult to make my references to identified issues. The manuscript needs to undergo grammar checking and sentence construction.

**Introduction**

1. “Given this significant functional disability prevalence among older people in Ghana,”

*The use of the word “significant” in this sentence is not justified, given the context in which it was used. A much milder term may be used.*

2. “the extent to which cohesive neighbourhood associate with functional disability of older persons and the nature of the relationship, is unexplored in Ghana”

*The use of the word “unexplored” should be revised. There are works relating to the subject under investigation.*

3. *In text citation number 17, should be written properly.*
4. In low-and middle-income countries including Ghana

*A comma (,) should be placed after Ghana*

5. In low-and middle-income countries including Ghana evidence on neighbourhood social cohesion and its measures, and functional disability among older people is scant.

*Again, much milder constructions should be considered. There are works in this area.*

**Methods**

**Physical activity**

1. “the overall PA”

*Start with a capital T*

**Results**

1. *The significance level values were not stated in the writing of the results. E.g., (OR=0.94, 95%CI: 0.93, 0.94). Only the odds ratio and confidence intervals were stated. Authors can add the P values. Also, I am aware the adjusted odds ratios are written as “AOR”. Authors should kindly check in on this. These should be updated in the abstract results also.*
2. That is, participating in in community activities and report engaging in physical activity by older adults were 2% less likely to experience functional disability (OR, 0.98, CI: 0.98, 0.99).

*There are typos in the sentence. Also, I do not seem to follow the 2% less likelihood from the stated results (OR, 0.98, CI: 0.98, 0.99).*

3. *Kindly check the reporting of your regression results again.*

**Discussion**

1. The current study finding that a more perceived socially cohesive neighbourhood was associated with a lower functional disability among older people confirms available studies in other low- and middle-income countries [33-35].

*There are typos in the sentence. Also, Reference number 34 says something about Japanese. I do not think Japan is a low- and middle-income country. Please check your reference.*
