# Supplementary material for: Perceived neighborhood social cohesion and functional disability among older adults: The moderating roles of sex, physical activity, and multi-morbidity
Source: PLoS One. 2024 Jan 31;19(1):e0293016. doi: 10.1371/journal.pone.0293016 (PMC10830004; doi:10.1371/journal.pone.0293016)
Supplement: Attachment — Submitted filename: Responses to Author.docx [file pone.0293016.s013.docx]

**Responses to Author**

| **Reviewer 1** | **Reviewer Comments** | **Our Response** |
| --- | --- | --- |
|  | 1. This is a well written article, and I really enjoyed reading it. It will be a worthwhile addition to the journal. The authors' compelling Introduction argues for the necessity of their study. In general, the methods used were clearly described. The authors' presentation of the findings is engaging and convincing. The results were integrated into the more general literature during the discussion. Findings and recommendations were explicit. However, the authors could improve the paper by considering some minor points/ suggestions which can be looked at. | We appreciate this complement. Authors have responded to all comments. |
|  | 2. Page 2. Introduction: line 6. It seems there is omission of a word in: “due to its influential role on the wellbeing older people” | This sentence has been corrected. |
|  | 3. Methods - Sample size: Page 5. Line 1 Authors did not provide any justification for the use of the instrument and also did not provide evidence of the regions where the sample size (4406) were drawn from in Ghana. | The sentence “The Study on global AGEing and adult health (SAGE) Ghana Wave 2 data, which was collected within two years (2014-2015) across all regions of Ghana was used in this study” has been provided indicating that it was a countrywide study. The details and reason for adopting multistage cluster sampling has been explained by the paper references. |
|  | 4. Page7, line 12; It is not clear why “pedal cycle” was categorized as walk. The question is what type of walk? Since the energy expenditure in both activities are not the same unless walk is properly defined. | It is true that the energy expenditure from these two activities are not the same, however, in this study we classified walk to represent any participant who reported taking part of any of this activities. Therefore, we interpreted the findings in relation to this measurement. |
|  | 5. page 9-10 (Tables). Authors did not provide any Table /link to how PA was measured | We explained under “Physical activity” how PA was measured. PA was measured with walking, moderate activity, vigorous activity. |
|  | 6. Page 10 Tables. Authors should be consistence with the use of sex and gender. Though sex was used throughout the work, gender was used in the tables instead | We have replaced “gender” with “sex” throughout. |
|  | 7. Page 13. Paragraph 1 line 11. The last sentence. “Lastly there is the need to promote PA……” It will be appropriate if authors provide measures or strategies that can be adopted to promote older people’s participation in PA. authors should also provide the agency or body to which this recommendation is been address to. | The sentence now reads “Lastly, social workers can use the media to promote the need for physical activity in old age for a desired functional health and quality of life. Social workers working within communities can sensitize older adults and families on the need to incorporate physical activity in their daily activities”. |
|  |  |  |
|  |  |  |
| **Reviewer 2** | There were no line numbers in the document. Thus, it made it quite difficult to make my references to identified issues. | We have included line numbers in the manuscript. |
|  | The manuscript needs to undergo grammar checking and sentence construction. | We have done a thorough editing of the manuscript and checked for grammar as much as possible. |
| Introduction | 1. “Given this significant functional disability prevalence among older people in Ghana,”   *The use of the word “significant” in this sentence is not justified, given the context in which it was used. A much milder term may be used.* | We have revised the sentence as:  Given this **emerging** functional disability prevalence among older people in Ghana, they may rely on informal social networks and other organized events to overcome daily life demands, including dealing with functional disability. |
|  | 2. “the extent to which cohesive neighbourhood associate with functional disability of older persons and the nature of the relationship, is unexplored in Ghana”   *The use of the word “unexplored” should be revised. There are works relating to the subject under investigation.* | We have revised the sentence to:  There is very little understanding of the extent to which cohesive neighbourhood is associated with the functional disability of older persons and the nature of the relationship in Ghana. |
|  | 3. In text citation number 17, should be written properly. | corrected |
|  | 4. In low-and middle-income countries including Ghana   *A comma (,) should be placed after Ghana* | Corrected. |
|  | 5. In low-and middle-income countries including Ghana evidence on neighbourhood social cohesion and its measures, and functional disability among older people is scant.   *Again, much milder constructions should be considered. There are works in this area.* | The sentence has been reworded:  There is limited research on the relationship between neighborhood social cohesion and functional disability among older people in low- and middle-income countries such as Ghana. |
| Method | 1. “the overall PA”   *Start with a capital T* | Corrected – See line 182 |
| Results | 1. *The significance level values were not stated in the writing of the results. E.g., (OR=0.94, 95%CI: 0.93, 0.94). Only the odds ratio and confidence intervals were stated. Authors can add the P values. Also, I am aware the adjusted odds ratios are written as “AOR”. Authors should kindly check in on this. These should be updated in the abstract results also.* | P values have been provided at respective places. All adjusted odds ratios have been written us “aOR” |
|  | 2. That is, participating in in community activities and report engaging in physical activity by older adults were 2% less likely to experience functional disability (OR, 0.98, CI: 0.98, 0.99).   *There are typos in the sentence. Also, I do not seem to follow the 2% less likelihood from the stated results (OR, 0.98, CI: 0.98, 0.99).* | The sentence has been reworded:  Even among the three moderators, only physical activity moderated the association between perceived community-level participation and functional disability. Older people participating in community and physical activities were 2% less likely to experience functional disability (OR, 0.98, CI: 0.98, 0.99) (see S5 Table). |
|  | 3. *Kindly check the reporting of your regression results again.* | We have checked and can confirm that is correct. |
| Discussion | 1. The current study finding that a more perceived socially cohesive neighbourhood was associated with a lower functional disability among older people confirms available studies in other low- and middle-income countries [33-35].   *There are typos in the sentence. Also, Reference number 34 says something about Japanese. I do not think Japan is a low- and middle-income country. Please check your reference.* | The sentence has been corrected |
